# Cell-Mediated Degradation Regulates Human Mesenchymal Stem Cell Chondrogenesis and Hypertrophy in MMP-Sensitive Hyaluronic Acid Hydrogels

**DOI:** 10.1371/journal.pone.0099587

**Published:** 2014-06-09

**Authors:** Qian Feng, Meiling Zhu, Kongchang Wei, Liming Bian

**Affiliations:** 1 Division of Biomedical Engineering, the Chinese University of Hong Kong, Hong Kong, the People's Republic of China; 2 Department of Mechanical and Automation Engineering and the Shun Hing Institute of Advanced Engineering, the Chinese University of Hong Kong, Hong Kong, the People's Republic of China; 3 The Chinese University of Hong Kong Shenzhen Research Institute, the People's Republic of China; University of California, San Diego, United States of America

## Abstract

Photocrosslinked methacrylated hyaluronic acid (MeHA) hydrogels support chondrogenesis of encapsulated human mesenchymal stem cells (hMSCs). However, the covalent crosslinks formed via chain polymerization in these hydrogels are hydrolytically non-degradable and restrict cartilage matrix spatial distribution and cell spreading. Meanwhile, cells are known to remodel their surrounding extracellular matrix (ECM) by secreting catabolic enzymes, such as MMPs. Hydrogels that are created with bifunctional crosslinkers containing MMP degradable peptide sequences have been shown to influence hMSC differentiations. However, crosslinks formed in the MMP-degradable hydrogels of these previous studies are also prone to hydrolysis, thereby confounding the effect of MMP-mediated degradation. The objective of this study is to develop a MMP-sensitive but hydrolytically stable hydrogel scaffold and investigate the effect of MMP-mediated hydrogel degradation on the chondrogenesis of the encapsulated hMSCs. Hyaluronic acid macromers were modified with maleimide groups and crosslinked with MMP-cleavable peptides or control crosslinkers containing dual thiol groups. The chondrogenesis of the hMSCs encapsulated in the hydrolytically stable MMP-sensitive HA hydrogels were compared with that of the MMP-insensitive HA hydrogels. It was found that hMSCs encapsulated in the MMP-sensitive hydrogels switched to a more spreaded morphology while cells in the MMP-insensitive hydrogels remained in round shape. Furthermore, hMSCs in the MMP-sensitive hydrogels expressed higher level of chondrogenic marker genes but lower level of hypertrophic genes compared to cells in the MMP-insensitive hydrogels. As a result, more cartilage specific matrix molecules but less calcification was observed in the MMP-degradable hydrogels than in the MMP-insensitive hydrogels. Findings from this study demonstrate that cell-mediated scaffold degradation regulates the chondrogenesis and hypertrophy of hMSCs encapsulated in HA hydrogels.

## Introduction

Methacrylated hyaluronic acid (MeHA) hydrogels provide a stable 3D environment that is conducive to the chondrogenesis of human mesenchymal stem cells (hMSCs) and neocartilage formation [1]. However, the covalently crosslinked MeHA hydrogels undergo minimal degradation by hydrolysis after extended incubation in aqueous environment. As a result, hydrogels fabricated with high MeHA concentrations hinder the spatial distribution of cartilaginous matrix synthesized by the encapsulated hMSCs. An ideal scaffold material for tissue engineering should be able to degrade in concert with the matrix elaboration by the seeded cells. Numerous studies have attempted to develop scaffolds that can degrade either by hydrolytic or enzymatic actions. Cells are known to remodel their surrounding extracellular matrix (ECM) by secreting catabolic enzymes, such as matrix metalloproteinases (MMPs). Hydrogels that are designed to be sensitive to MMP actions have been demonstrated by several previous studies. These studies also revealed that cell-mediated scaffold degradation substantially impacts the differentiation of mesenchymal stem cells (MSCs) [2]–[5]. For example, a recent study demonstrated that the MMP-sensitive PEG (polyethylene glycol) hydrogels promoted the chondrogenesis of encapsulated human MSCs (hMSCs) [6]. However, few studies have examined the effect of cell-mediated matrix degradation on hMSC chondrogenesis and subsequent hypertrophy in hyaluronic acid hydrogels. Furthermore, many of the previous investigations used MMP-cleavable peptides containing dual cysteine residues to crosslink acrylated macromers [4], [5]. The resulting hydrolytically degradable thiol-ether-ester bonds confound the effect of enzymatic degradation of the hydrogels by cell-secreted MMPs.

Chondrogenically induced MSCs display the propensity to continue differentiating towards a hypertrophic phenotype, resulting in extensive calcification of the ECM after ectopic transplantation [7], [8]. This process is similar to that observed in the terminal differentiation of hypertrophic chondrocytes in the growth plate and articular cartilage, where the components and structures of the ECM regulate chondrocyte hypertrophy and matrix calcification [9]–[11]. Reciprocally, hypertrophic chondrocytes produce increased MMPs such as MMP13 to remodel their surrounding cartilage matrix in a way that facilitates the ensuing mineralization [12]. Therefore, the dynamic interplay between the degradational properties of the scaffold materials and MMPs synthesis by chondrogenically induced hMSCs may influence hypertrophic differentiation of these MSCs, and consequently the calcification of the neocartilage matrix.

Previously we showed that the crosslinking densities of chondrocytes or hMSC-laden HA hydrogels influence neocartilage formation, subsequent hypertrophy and neocartilage calcification [13], [14]. In addition, a recent study demonstrated that MMP-sensitive but hydrolysis-insensitive HA hydrogels can be fabricated by crosslinking maleimide modified HA macromers with MMP-cleavable peptides [2]. However, further understanding on the regulation of hMSCs hypertrophy and tissue calcification is needed to ensure successful clinical application of hMSCs for cartilage repair. We hypothesize that MMP-sensitive HA hydrogels will regulate the chondrogenic and hypertrophic differentiation of the encapsulated hMSCs. Thus, the objective of this study is to evaluate the effect of cell-mediated degradation of HA hydrogels on the chondrogenesis and hypertrophy of encapsulated hMSCs and the neocartilage calcification using an *in vitro* MSC hypertrophy model [15].

## Material and Methods

### Macromer synthesis

**Figure 1:**
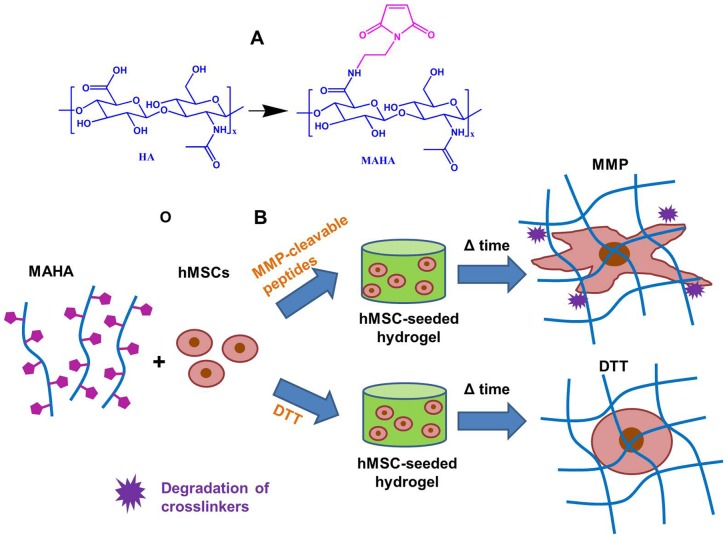
The chemical structure of the maleimide modified HA (MAHA) and encapsulation of hMSCs in MAHA hydrogels. (A) Modification of hyaluronic acid to yield maleimide modified HA (MAHA). (B) Encapsulation of hMSCs in MAHA hydrogels using either MMP-cleavable peptides or MMP-insensitive DTT as crosslinkers.

Maleimide modified HA (MAHA) was synthesized as previously reported [2] (**Figure 1** A). Briefly, tetrabutylammonium salt of HA (HA-TBA) was first synthesized by reacting sodium hyaluronate with ion exchange resin Dowex-100 and neutralized with TBA-OH. Lyophilized HA-TBA was reacted with N-(2-Aminoethyl) maleimide trifluoroacetate salt in DMSO in the presence of benzotriazole-1-yl-oxy-tris-(dimethylamino)-phosphonium hexafluorophosphate (BOP, Sigma) at room temperature under nitrogen with stirring for 2 hours followed by dialysis at 4°C and lyophilization. The final macromer products were confirmed by ^1^H NMR to have a maleimide modification level of ∼30%.

### Mechanical testing

After 24 hours of equilibration in PBS (phosphate buffered saline), acellular HA hydrogels were first equilibrated in creep to a tare load of 2 gram by an impermeable loading platen in a loading chamber filled with PBS. From this offset, stress relaxation tests were performed with a single compression ramp at a speed of 10%/min until reaching 10% strain. The equilibrium Young's modulus (E_Y_) was determined by the equilibrium load obtained after 1000 sec of relaxation under unconfined compression at 10% strain.

### Sample preparation and in vitro culture

Passage 3 human MSCs (Lonza) were encapsulated in 1.5 wt% MAHA hydrogel constructs (20 million cells/ml, Ø5 mm, 2.5 mm thickness) using MMP cleavable peptides (GCRDVPMS↓MRGGDRCG, cysteine residues included for crosslinking maleimides, ↓ indicates MMP cleavage site) or MMP insensitive dithiothreitol (DTT group) as crosslinkers (**Figure 1** B). The molar ratio between the maleimides on the MAHA and the thiols of the crosslinkers was fixed at 2∶1 so that 50% of the maleimides were consumed for crosslinking. Constructs were cultured in chondrogenic media (DMEM, 1% ITS+Premix, 50 µg/ml L-proline, 0.1 µM dexamethasone, 0.9 mM sodium pyruvate, 50 µg/ml ascorbate, antibiotics) supplemented with TGF-β3 (10 ng/ml) for 14 days and then in hypertrophy induction media (1 nM dexamethasone, 1 nM triiodothyronine and 10 mM β-glycerophosphate) for an additional 14 days [15]. Cell viability was assessed using the LIVE/DEAD Assay Kit (Molecular Probes) where live cells were stained green with calcein-AM and dead cells stained red with ethidium homodimer.

### Gene expression analysis

For gene expression analysis, samples were homogenized in Trizol Reagent (Invitrogen) with a tissue grinder, RNA was extracted according to the manufacturer's instructions, and the RNA concentration was determined using an ND-1000 spectrophotometer (Nanodrop Technologies). One microgram of RNA from each sample was reverse transcribed into cDNA using reverse transcriptase (Superscript II, Invitrogen) and oligoDT (Invitrogen). Polymerase chain reaction (PCR) was performed on an Applied Biosystems 7300 Real-Time PCR system using Taqman primers and probes specific for GAPDH (housekeeping gene) and other genes of interest. Sequences of the primers and probes used are listed in **Table 1**. The relative gene expression was calculated using the ΔΔC_T_ method, where fold difference was calculated using the expression 2^ΔΔCt^. Each sample was internally normalized to GAPDH and each group was normalized to the expression levels of MSCs at the time of encapsulation (i.e., after expansion and before differentiation). Relative expression levels greater than 1 represent up-regulation with culture, while relative expression levels less than 1 represent down-regulation of that gene compared to that of initially encapsulated MSCs.

**Table 1:** Sequences of primers and probes used for Real-Time PCR. Sequences related to gene type X collagen and MMP13 are proprietary to Applied Biosystems Inc. and not disclosed.

Gene	Forward Primer	Reverse Primer	Probe
GAPDH	AGGGCTGCTTTTAACTCTGGTAAA	GAATTTGCCATGGGTGGAAT	CCTCAACTACATGGTTTAC
COL II	GGCAATAGCAGGTTCACGTACA	CGATAACAGTCTTGCCCCACTT	CTGCACGAAACATAC
Aggrecan	TCGAGGACAGCGAGGCC	TCGAGGGTGTAGCGTGTAGAGA	ATGGAACACGATGCCTTTCACCACGA

### Biochemical analysis

One-half of each construct was weighed wet, lyophilized, reweighed dry, and digested in 0.5 mg/ml Proteinase-K (Fisher Scientific) at 56°C for 16 hrs. The PicoGreen assay (Invitrogen, Molecular Probes) was used to quantify the DNA content of the constructs with Lambda phage DNA (0–1 mg/ml) as a standard [16]. For each sample, both the mass of the entire gel and the half gel used for DNA assay were measured. The total amount of DNA per sample was calculated by scaling the amount of DNA detected in the half gel by a weight ratio (total weight/half weight). The GAG content was measured using the dimethylmethylene blue (DMMB, Sigma Chemicals) dye-binding assay with shark chondroitin sulfate (0–50 mg/ml) as a standard [17]. The overall collagen content was assessed by measuring the orthohydroxyproline (OHP) content via dimethylaminobenzaldehyde and chloramine T assay. Collagen content was calculated by assuming a 1∶7.5 OHP-to-collagen mass ratio [18]. The collagen and GAG contents were normalized to the disk wet weight. Calcium content of the samples was analyzed using a calcium quantification kit (Biovision).

### Histological analysis

The remaining halves of the constructs were fixed in 4% formalin for 24 h, embedded in paraffin, and processed using standard histological procedures. The histological sections (8 µm thick) were stained for targets of interest using the Vectastain ABC kit and the DAB Substrate kit for peroxidase (Vector Labs). Briefly, sections were predigested in 0.5 mg/ml hyaluronidase for 30 min at 37°C and incubated in 0.5 N acetic acid for 4 h at 4°C to swell the samples prior to overnight incubation with primary antibodies at dilutions of 1∶100, 1∶200, and 1∶3 for chondroitin sulfate (mouse monoclonal anti-chondroitin sulfate, Sigma), and type I (mouse monoclonal anti-collagen type 1, Sigma) and type II collagen antibodies (mouse monoclonal anti-collagen type II, Developmental Studies Hybridoma Bank), respectively. Non-immune controls underwent the same procedure without primary antibody incubation.

### Statistical analysis

All data are presented as mean ± standard deviation. Statistica (Statsoft, Tulsa, OK) was used to perform statistical analyses using two-way ANOVA, followed by Tukey's HSD post hoc testing to allow comparison between groups (n = 4 samples per group), with culture duration and experimental group as independent factors.

## Results

### Characterization of acellular hydrogels

**Figure 2:**
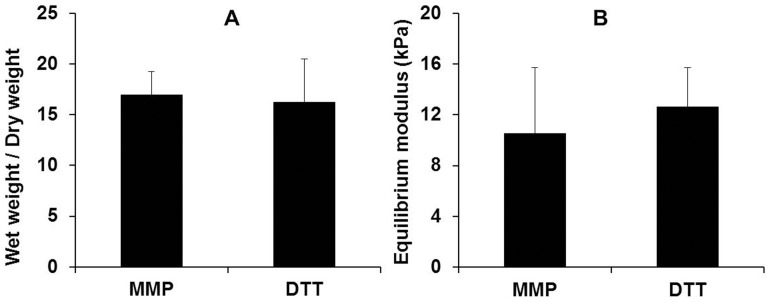
The physical properties of the hydrogels formed using different crosslinkers. (A) Weight degree of swelling (the ratio between the swollen wet weight and dry weight) and (B) equilibrium modulus of acellular hydrogels crosslinked using either MMP-cleavable peptides or MMP-insensitive DTT after 24 hours of equilibration in phosphate buffered saline.

MMP-cleavable peptides or DTT were used as crosslinkers to consume 50% of the maleimide groups conjugated on the HA macromers to form acellular hydrogels. After 24 hours of equilibration in phosphate buffered saline, the weight degree of swelling (the ratio between the swollen wet weight and dry weight) of the acellular hydrogels is similar between the MMP-cleavable peptides and DTT crosslinked hydrogels (**Figure 2A**). There is no significant difference in the equilibrium modulus of these two groups of acellular hydrogels, either (**Figure 2B**).

### Cell morphology and viability of the hMSCs encapsulated

**Figure 3:**
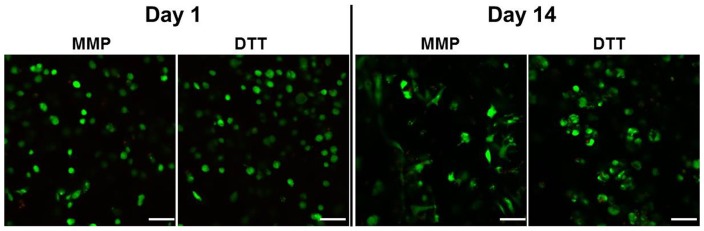
The cell viability staining on day 1 (A) and 14 (B) of the culture. Bar  = 100 µm.

On the first day of culture, cells in both MMP-sensitive (MMP) and MMP-insensitive (DTT) hydrogels exhibit a round morphology. However, after 2 weeks of culture, hMSCs encapsulated in MMP-sensitive HA hydrogels (MMP) switched from the initial round morphology to a more spreaded profile, whereas those cells in the control DTT crosslinked hydrogels (DTT) remained in the spherical shape (**Figure 3**). Cell viability staining indicated that the majority of the cells (>90%) in both groups of hydrogels remained viable after 2 weeks of culture (**Figure 3**).

### Expression of chondrogenic and hypertrophic marker genes

**Figure 4:**
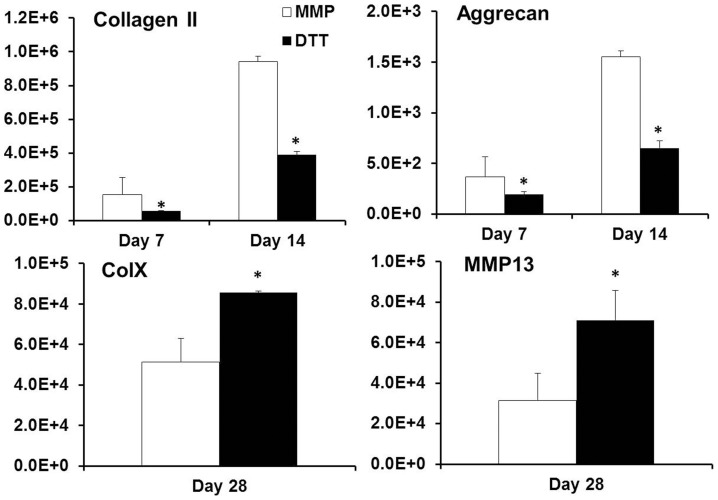
The gene expression of type II collagen, aggrecan, type X collagen and MMP13 in fold changes on day 7, 14 or 28 of the culture normalized to pre-differentiated cells. *p<0.05 vs. MMP group (n = 4).

mRNA expression of chondrogenic markers like type II collagen and aggrecan was significantly up-regulated in the MMP-sensitive hydrogels (MMP group) compared to the DTT group (**Figure 4**). For example, on day 7 and 14 of the culture the type II collagen expression of the MMP group was 177% and 142% higher than that of the DTT group, respectively, while the aggrecan expression of the MMP group was 89% and 132% higher, respectively. In contrast, hypertrophy marker genes including MMP13 and type X collagen was significantly down regulated in the MMP degradable hydrogels on day 28 in the hypertrophy induction media (**Figure 4**). The MMP13 and type X collagen mRNA level of the MMP group was 44% and 59% of that of the DDT group.

### Biosynthesis of cartilaginous matrix in the hMSC-seeded hydrogels

**Figure 5:**
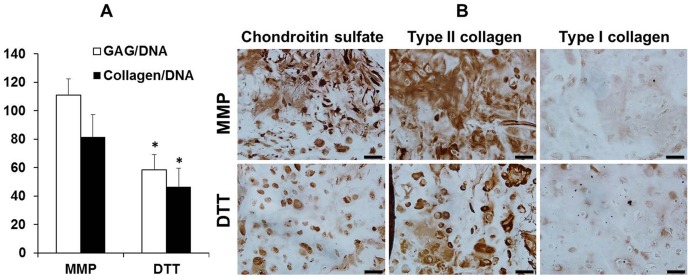
The analysis of the cartilaginous matrix contents of the hMSC-seeded hydrogels. GAG and collagen content (normalized to the DNA content) of hMSC-laden HA hydrogels (A) and immunohistochemistry staining of the hydrogel constructs against chondroitin sulfate, type II and I collagen after 28 days of culture (B), Bar  = 50 µm, *p<0.05 vs. MMP group (n = 4).

The hMSC-seeded MMP-sensitive hydrogels also developed significantly higher contents of cartilaginous matrix molecules such as GAG (glycosaminoglycan) and collagens after 28 days of culture (**Figure 5** **A**). To be specific, when normalized by the DNA content, the hMSCs in the MMP-sensitive hydrogels produced 90% and 76% more GAG and collagen compared to the cells in MMP-insensitive hydrogels. Immunohistochemical staining revealed more intense staining against chondroitin sulfate and type II collagen, the two prominent cartilage matrix components, in the hydrogels crosslinked with MMP-cleavable peptides compared to the hydrogels crosslinked with DTT (**Figure 5** **B**). Type I collagen staining was minimal indicating that the majority of the collagens produced by hMSCs was cartilaginous type II collagen (**Figure 5** **B**).

### Hypertrophic calcification of the hMSC-seeded hydrogels

**Figure 6:**
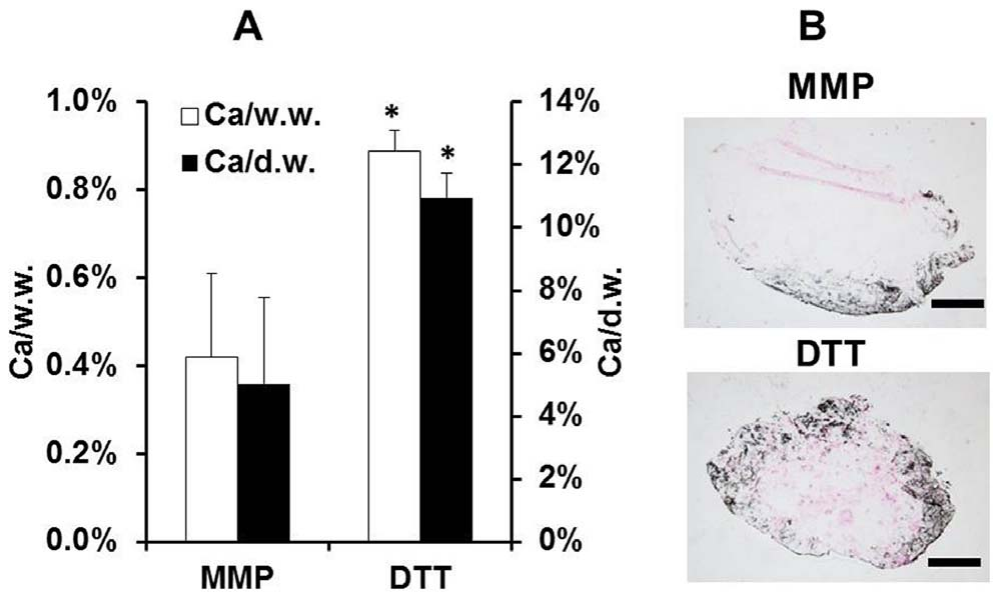
The evaluation of the hypertrophic calcification of the hydrogels. Calcium content of the hMSC-laden hydrogels normalized to wet weight (w.t.) and dry weight (d.w) (A); and Von Kossa staining of the histological sections of the hydrogels after 28 days of culture, bar  = 500 µm, *p<0.05 vs. MMP group (n = 4).

After 28 days of the culture, the calcium content of the MMP degradable gels was lower than that of the MMP insensitive gels as evidenced by calcium quantification assay (53% or 55% lower when normalized to the wet or dry weight of the sample, respectively) (**Figure 6** **A**). Von Kossa staining showed more staining in the DTT group compared to the MMP group confirming this finding (**Figure 6** **B**).

## Discussion

This study demonstrate that hMSCs encapsulated in MMP-degradable/sensitive HA hydrogels are capable of remodeling the surrounding hydrogel structures and spreading within the hydrogels. Furthermore, this cell-mediated hydrogel degradation enhanced chondrogenesis and suppressed hypertrophy of the encapsulated hMSCs. This led to the increased cartilage matrix deposition and reduced calcification in the MMP-degradable hydrogels (MMP) compared to the non-degradable control hydrogels (DTT) after long term culture.

In this study, an *in vitro* hypertrophy model was used to evaluate hMSC hypertrophy after chondrogenic induction [15]. This model allows us to examine the influence of cell-mediated degradation on hMSC hypertrophy in a defined *in vitro* setting, thereby avoiding the systemic complexity and considerable expense of *in vivo* studies. Switching to the hypertrophy induction media after 2 weeks of chondrogenic induction not only expedites the hypertrophic differentiation of hMSCs by eliminating the hypertrophy-suppressing TGF-β3 but also provides the necessary phosphate donors for mineralization. Therefore, several previous studies employed this *in vitro* model to study hMSC hypertrophy [15], [19].

It is known that hydrogels fabricated using methacrylated hyaluronic acid (MeHA) experience little hydrolytic degradation over extended period likely due to the methyl groups in the methacrylates, which shield water molecules by steric hindrance [20]. Our previous study indicated that high crosslinking density of MeHA hydrogels resulted in reduced cartilage matrix biosynthesis and more restricted cartilage matrix distribution [14]. The hypertrophic differentiation of the hMSCs and ensuing neocartilage calcification was also enhanced in highly crosslinked MeHA hydrogels compared to hydrogels of low crosslinking density [14]. Prior studies showed that MMP-sensitive PEG hydrogels promoted neocartilage development by the encapsulated chondrocytes and MSCs [6], [21]. Compared with PEG hydrogels, HA hydrogels afford a more favorable microenvironment for chondrogenesis by providing binding sites for cell surface receptors such as CD44 and CD168, activation of which are important to chondrogenesis [22]–[24]. This study confirmed that MMP-sensitive HA hydrogels also enhanced chondrogenesis of the encapsulated hMSCs. The enhanced cell spreading and motility in the MMP-sensitive hydrogels allowed more cell-cell interactions, which are essential to chondrogenesis [25], [26]. The proteolytic action of MMPs also releases ECM bound growth factors that are inducer of chondrogenesis [27]. These may have contributed to the enhanced chondrogenesis of the hMSCs in the MMP-sensitive HA hydrogels.

Importantly, prior design of MMP-sensitivity in HA hydrogels was achieved by crosslinking acrylated HA with crosslinkers containing dual thiols [5]. The resulting thiol-ether-ester bonds are potentially labile to hydrolytic degradation. A separate unpublished study revealed that the acrylated HA (60% acrylate modification, which is even higher than the maleimide modification used in this study) hydrogels quickly degraded and lost the structural integrity within a week of culture. This not only makes the acrylated HA hydrogel unsuitable for long term cell culture studies but also confounds the effect of MMP-mediated degradation on hMSCs. In contrast, maleimides react with thiols to form stable thioether bonds [28], [29]. A prior study demonstrated that maleimide modified HA (MAHA) macromers can be crosslinked with the same thiol containing crosslinkers to form hydrogelsthat are resistant to hydrolytic degradation [2]. In contrast to the acrylated HA hydrogels, maleimide modified HA hydrogel constructs used in this study largely maintained their initial cylindrical morphology during the 4 weeks of culture without significant bulk degradation. Therefore, the results of this study clearly show that the cell-mediated HA hydrogel degradation via MMPs is indeed responsible for the enhanced chondrogenesis of the encapsulated hMSCs.

This study also revealed that the hMSCs encapsulated in the MMP-sensitive hydrogels displayed diminished hypertrophic behaviors leading to reduced matrix calcification. One likely explanation to this finding is the different amount of cartilaginous matrix deposition in the hydrogels. The MMP-sensitive hydrogels accumulated more GAG and collagen than the MMP-insensitive hydrogels (**Figure 5**). The two major cartilage ECM molecules, proteoglycans and type II collagen, are known to promote and stabilize chondrogenic phenotype and thereby suppress matrix calcification [11], [30], [31]. Proteoglycans also bind and stabilize various growth factors and help maintain prolonged bioactivity of growth factors [32], [33]. Some of these growth factors such as TGF-βs are inhibitors of hypertrophic differentiation [34]. Therefore, the higher cartilage matrix content in the MMP-sensitive hydrogels may have contributed to the suppression of hMSC hypertrophy.

A previous study by another group shows that longer therefore more complete chondrogenic induction actually expedites hypertrophy of chondrogenically induced MSCs compared to shorter chondrogenic induction [35]. Our previously published work also indicates that compared to poorly differentiated cells, more thoroughly differentiated hMSCs result in more significant hypertrophy after *in vivo* implantation [7]. The correlation between the extent of MSCs chondrogenesis and the propensity of subsequent hypertrophy certainly warrants further investigation. Nevertheless, multiple factors can regulate hypertrophy at the same time. A study also shows that activated RhoA/ROCK signaling, which is associated with cell spreading and cytoskeletal tension, inhibits chondrocyte hypertrophy [36]. We postulate that the spreading of the differentiated hMSCs in the MMP-sensitive hydrogels may have inhibited hypertrophy in this case.

The MMP-cleavable peptide sequence used in this study is a target of multiple MMP species [37]. Therefore, even though the MMP13 expression in the MMP-sensitive hydrogels was lower than that in the MMP-insensitive hydrogel, cells were still able to break down the MMP-cleavable crosslinks. However, the exact mechanisms underlying the observed effects of cell-mediated degradation on hMSC chondrogenesis remain to be elucidated. Future study is needed to better understand the mechanism of this regulation of hMSC chondrogenic differentiation by cell-mediated scaffold degradation.

In the MMP-sensitive hydrogels, significant cell spreading was only observed after around 10 days of culture. The serum-free media used in this study likely induced less cell MMP synthesis than serum-supplemented media do, in which cell spreading in similar MMP-sensitive hydrogels were observed at earlier time points in an unpublished separate study. It should also be noted that significant variation in MMP expression exists among hMSCs from different donors. The hMSCs used in this study are from a healthy young donor. Our previous experiments revealed that hMSCs from osteoarthritic patients express significantly higher MMPs and would have degraded these HA hydrogels faster.

Previous studies suggest that a round cell morphology and disrupted cell cytoskeletal structure is closely associated with chondrogenesis [38]–[40]. This appears conflicting to our finding that the more spread hMSCs in the MMP-sensitive hydrogels exhibited better chondrogenesis than the rounded hMSCs in the control group. However, most of these previous reports so far indicate that an early disruption of the cytoskeletal structure resulting in round cell morphology promote chondrogenesis. As mentioned above, hMSCs started significant spreading after around 10 days of culture in the MMP-sensitive hydrogels. Our previous published studies revealed significant upregulation of chondrogenic marker genes including sox9, collagen type II and aggrecan in hMSCs after only 3–5 days of induction using TGF-β3 [7], [24]. We hypothesize that the majority of the hMSCs in the MMP-sensitive hydrogels had already moved down along the chondrogenic differentiation pathway before they started to spread in the hydrogels. Therefore, the cell spreading had little effect on these fully differentiated hMSCs. Further examination on the effect of cytoskeletal structure and cell shape on chondrogenic phenotype of the differentiated stem cells is needed.

A limitation of this study is that the two bifunctional crosslinkers used, namely, the MMP-cleavable peptide and DTT, are of different length and chemical structures. Therefore, even though the two groups of hydrogels exhibited similar level of swelling and mechanical stiffness and will likely have comparable permeability as well, the effect of different crosslinker properties as a confounding factor cannot be ruled out. Future study will explore the use of control crosslinkers that are structurally stable, biochemically inert and yet of similar length as that of the MMP-cleavable peptides.

## Conclusions

This study revealed that cell-mediated degradation via MMPs in hyaluronic acid hydrogels enhanced chondrogenesis but suppressed hypertrophy of hMSCs. Our utilization of a hydrolytically stable HA hydrogel eliminates the confounding factor of hydrogel degradation due to hydrolysis. Knowledge obtained from this study will help guide the design and optimization of HA hydrogels as carrier materials for cartilage repair using hMSCs.
